# Actin‐related protein 5 suppresses the cooperative activation of cardiac gene transcription by myocardin and MEF2

**DOI:** 10.1002/2211-5463.13549

**Published:** 2023-01-12

**Authors:** Tsuyoshi Morita, Ken'ichiro Hayashi

**Affiliations:** ^1^ Department of Biology Wakayama Medical University Japan; ^2^ Department of Ophthalmology Yamaguchi University Graduate School of Medicine Japan; ^3^ Department of RNA Biology and Neuroscience Osaka University Graduate School of Medicine Japan

**Keywords:** actin‐related protein, ARP5, cardiomyopathy, MEF2, myocardin, MYOCD

## Abstract

MYOCD is a transcription factor important for cardiac and smooth muscle development. We previously identified that actin‐related protein 5 (ARP5) binds to the N‐terminus of MYOCD. Here, we demonstrate that ARP5 inhibits the cooperative action of the cardiac‐specific isoform of MYOCD with MEF2. ARP5 overexpression in murine hearts induced cardiac hypertrophy and fibrosis, whereas ARP5 knockdown in P19CL6 cells significantly increased cardiac gene expression. ARP5 was found to bind to a MEF2‐binding motif of cardiac MYOCD and inhibit MEF2‐mediated transactivation by MYOCD. RNA‐seq analysis revealed 849 genes that are upregulated by MYOCD‐MEF2 and 650 genes that are repressed by ARP5. ARP5 expression increased with cardiomyopathy and was negatively correlated with the expression of Tnnt2 and Ttn, which were regulated by cardiac MYOCD‐MEF2. Overall, our data suggest that ARP5 is a potential suppressor of cardiac MYOCD during physiological and pathological processes.

## Graphical abstract

We found that the overexpression of ARP5 in the murine hearts leads to cardiac hypertrophy and fibrosis. ARP5 binds to the MEF2‐binding motif (MBM) of cardiac MYOCD and inhibits the interaction between MYOCD and MEF2, thereby suppressing cardiac gene expression. ARP5 is highly expressed in fetal and failing hearts, suggesting the developmental and pathological importance of ARP5 in the cardiovascular system.

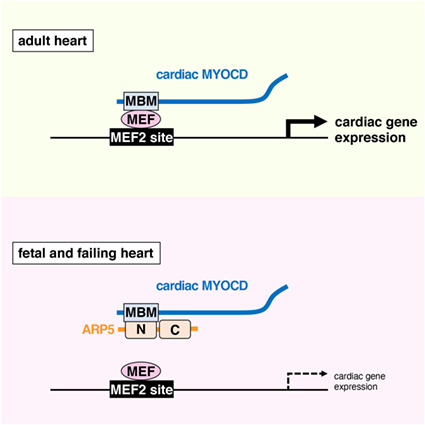

**Abbreviation**
AAV6: adeno‐associated virus serotype 6
ARP5: actin‐related protein 5
DMSO: dimethyl sulfoxide
DOX: doxycycline
GSEA: Gene Set Enrichment Analysis
MBM: MEF2‐binding motif
MEF2: myocyte enhancer factor 2
MKL: megakaryoblastic leukemia
MYOCD: myocardin
RPEL: RPxxxEL
SAP: SAF‐A/B, Acinus, PIAS
SRF: serum response factor

The myocardin family transcription factors, namely, myocardin (MYOCD), megakaryoblastic leukemia 1 (MKL1), and megakaryoblastic leukemia 2 (MKL2), are SAP (SAF‐A/B, Acinus, PIAS) domain family proteins that act primarily as transcriptional co‐activators of the serum response factor (SRF). MYOCD is specifically observed in the nucleus of cardiac and smooth muscle cells [1], whereas MKL1 and MKL2 are ubiquitously expressed and translocated from the cytoplasm to the nucleus upon activation [2, 3]. The N‐termini of these proteins contain two or three copies of the RPxxxEL motif (RPEL motif) to which monomeric actin (G‐actin) binds [2]. They are important sites for regulating the activity of MKL1 and MKL2. When G‐actin dissociates from the RPEL motifs, such as through actin polymerization, the nuclear localization signals of MKL1 and MKL2 are exposed, leading to their nuclear translocation and subsequent activation. In contrast, the actin‐binding affinity of the RPEL motifs is considerably lower in MYOCD; therefore, MYOCD is always localized to the nucleus [4]. Previously, we identified actin‐related protein 5 (ARP5), encoded by the *Actr5* gene, as a novel partner that binds to the N‐terminus of MYOCD instead of G‐actin [5]. Actin‐related proteins (ARPs) represent a family of proteins with high sequence similarity to actin, and ARP5 is one of the nuclear‐localized members [6]. In addition to the N‐domain and C‐domain, which share similarities with other ARPs and conventional actins, ARP5 has three unique domains in its N‐terminus (S1), central region (S2), and C‐terminus (S3) [5]. In addition to MYOCD, ARP5 binds to SRF via the S3 domain, thus preventing the MYOCD–SRF complex from binding to DNA. In vascular smooth muscle cells, *Actr5* expression increases with dedifferentiation, thereby suppressing the expression of smooth muscle genes that are regulated by the MYOCD–SRF complex [5].

During cardiac and smooth muscle development in mice, *Myocd* can be detected in the cardiac crescent at embryonic day 7.75 (E7.75) and the linear heart tube at E8.0 and then throughout the embryonic and postnatal heart [1]. *Myocd* is also expressed in the smooth muscle cells of the esophagus, aortic arch arteries, and pulmonary outflow tract at E13.5 [1]. *Myocd* knockout mice exhibit embryonic lethality before E9.5–E10.5 because of impaired cardiac morphogenesis and function and loss of differentiated vascular smooth muscle cells [7, 8]. Thus, MYOCD plays an important role in cardiac and smooth muscle differentiation. Creemers et al. [9] reported two tissue‐specific isoforms of MYOCD, myocardin‐856 in smooth muscle cells (smooth muscle MYOCD) and myocardin‐935 in cardiac cells (cardiac MYOCD). The cardiac *Myocd* gene consists of 14 exons and is translated from the first ATG codon, whereas the smooth muscle *Myocd* gene is translated from the second ATG codon in exon 4 because of an in‐frame stop codon present in the extra exon 2a. The longer cardiac MYOCD has three RPEL motifs, namely, RPEL1, 2, and 3, and a unique sequence for binding to the myocyte enhancer factor 2 (MEF2) protein (MEF2‐binding motif, MBM). In contrast, the shorter smooth muscle MYOCD lacks the RPEL1 and MBM motifs. Of the myocardin family proteins, only cardiac MYOCD directly binds to MEF2 and dramatically enhances its relatively weak transcriptional activity, which may be associated with cardiac‐specific gene expression by MYOCD.

In the present study, we demonstrated that ARP5 binds to the N‐terminus of cardiac MYOCD, not through RPEL motifs, but via a sequence near the MBM motif in a manner distinct from conventional actin. ARP5 competes with MEF2 to interact with cardiac MYOCD and suppresses the MYOCD‐MEF2‐mediated transactivation of cardiac gene expression. *Actr5* expression was reduced in half during cardiac maturation and increased during the cardiomyopathic process. The exogenous overexpression of ARP5 induced cardiac hypertrophy and fibrosis with decreased expression of SRF‐ and MEF2‐regulated cardiac genes. Thus, ARP5 may be an important regulator of cardiac function by interacting with cardiac MYOCD.

## Results

### ARP5 overexpression induces cardiac hypertrophy and fibrosis

**Fig. 1:**
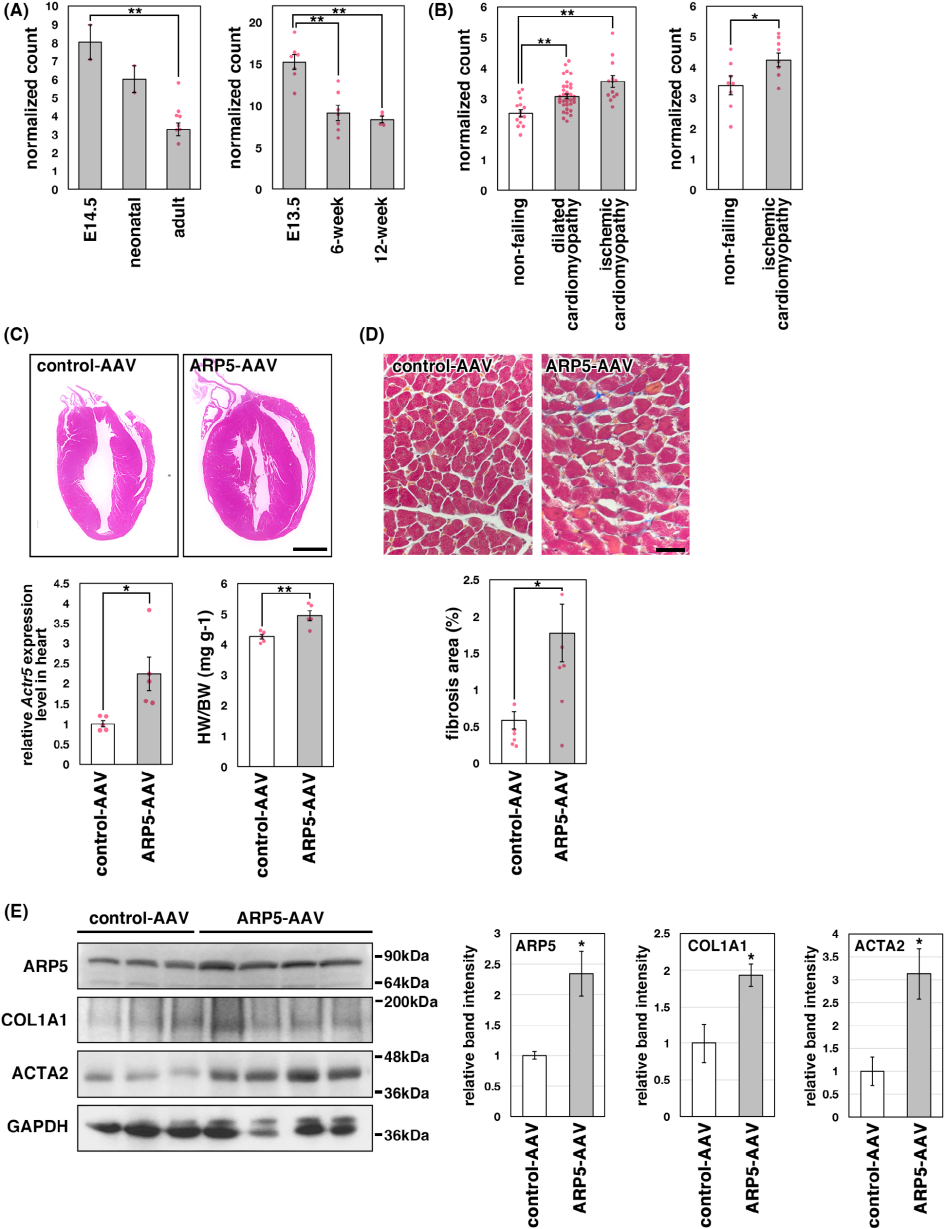
Change in Actr5 expression level in the hearts. (A) Changes in expression of the mouse *Actr5* gene during heart development. RNA‐seq data were obtained from the Gene Expression Omnibus (GEO) database (accession no. GSE79883, left panel; GSE58455, right panel). (B) Expression of human *ACTR5* gene in failing hearts. RNA‐seq data were obtained from GEO (GSE116250, left panel; GSE46224, right panel). (C) Hematoxylin and eosin staining images of murine hearts infected with control AAV6 (control‐AAV) or ARP5‐AAV6 (ARP5‐AAV) vectors (upper panels). Bar = 2 mm. *Actr5* expression in these hearts was measured by real‐time RT‐PCR (lower left panel). The ratio of the heart weight to body weight (HW/BW) was calculated and statistically analyzed (lower right panel). (D) Masson's trichrome staining images of control AAV6‐ and ARP5‐AAV6‐infected hearts (upper panels). Bar = 100 μm. The blue‐stained fibrotic areas were measured and statistically analyzed (lower panel). (E) Expression of ARP5, COL1A1, ACTA2, and GAPDH proteins in control AAV6‐ and ARP5‐AAV6‐infected hearts (left panel). The band intensities were measured and statistically analyzed (right panels). All statistical data in Fig. 1 are presented as the mean ± standard error of the mean (SEM). *P < 0.05, **P < 0.01 (Student's *t*‐test).

Previously, we reported that *Actr5* expression is decreased during smooth muscle and skeletal muscle differentiation [5, 10]. We evaluated the change in the expression of the *Actr5* gene during mouse heart development using RNA sequence (RNA‐seq) data from a public database and demonstrated that *Actr5* levels in adult hearts were reduced to approximately half of that in embryonic hearts (Fig. 1A). Furthermore, *Actr5* expression was increased in dilated and ischemic cardiomyopathic hearts (Fig. 1B). These data suggest that the expression of *Actr5* varies with the differentiation state of cardiac cells. To determine a role for ARP5 in the heart, the adeno‐associated virus serotype 6 (AAV6) vector was used to overexpress ARP5 in mouse hearts. AAV serotypes with various tissue‐specific tropisms have been identified, and AAV serotype 6 exhibits the highest transduction frequency in the heart [11]. ARP5‐AAV6 infection increased the expression of the *Actr5* gene in hearts by an average of approximately two‐fold compared with the mock AAV6 infection and resulted in a slight, but significant increase in relative heart weight (Fig. 1C). In addition, Masson's trichrome staining revealed increased collagen deposition in the enlarged hearts (Fig. 1D). Western analysis also showed the increased protein expression of type‐I collagen (COL1A1) and myofibroblast marker α‐smooth muscle actin (ACTA2) (Fig. 1E), indicating that cardiac fibrosis occurred in the ARP5‐AAV6‐infected hearts.

**Fig. 2:**
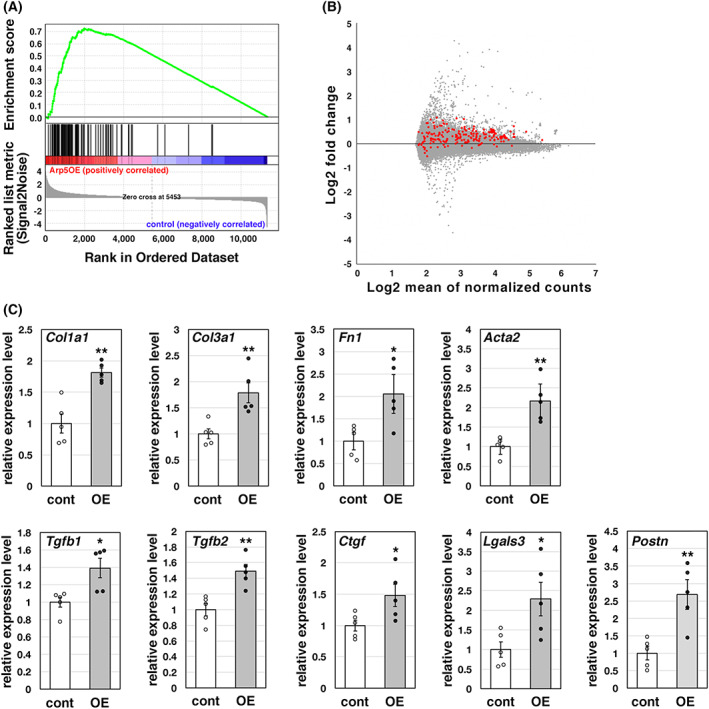
Upregulation of fibrotic genes in the ARP5‐overexpressing heart. (A) GSEA enrichment plot of the top 1000 genes whose expression was increased by ARP5‐AAV6 infection in the DNA microarray analysis for the gene set CUI_DEVELOPING_HEART_C3_FIBROBLAST_LIKE_CELL. (B) MA plot of the DNA microarray data between control AAV6‐ and ARP5‐AAV6‐infected hearts. The red dots indicate the position of the fibrotic genes. (C) Expression of fibrotic genes in control AAV6‐ (cont) and ARP5‐AAV6‐infected (OE) hearts was validated by real‐time RT‐PCR. Statistical data are presented as the mean ± standard error of the mean (SEM). *P < 0.05, **P < 0.01 (Student's *t*‐test).

We performed a DNA microarray analysis using RNA extracted from AAV6‐infected hearts, followed by a Gene Set Enrichment Analysis (GSEA) [12, 13]. The top 1000 genes upregulated by ARP5‐AAV6 infection were enriched in cardiac fibroblast genes (Fig. 2A). Increased expression of 195 out of 219 genes in the dataset ‘CUI_DEVELOPING_HEART_C3_FIBROBLAST_LIKE_CELL’ occurred in the ARP5‐AAV6‐infected hearts (Fig. 2B). Real‐time RT‐PCR confirmed the increased expression of pro‐fibrotic genes including *Col1a1*, *Col3a1*, *Fn1*, *Acta2*, *Tgfb*1, *Tgfb2*, *Ctgf*, *Lgals3*, and *Postn* (Fig. 2C). The data indicate that excessive ARP5 expression results in cardiac hypertrophy and fibrosis.

### ARP5 represses SRF‐ and MEF2‐regulated cardiac gene expression

**Fig. 3:**
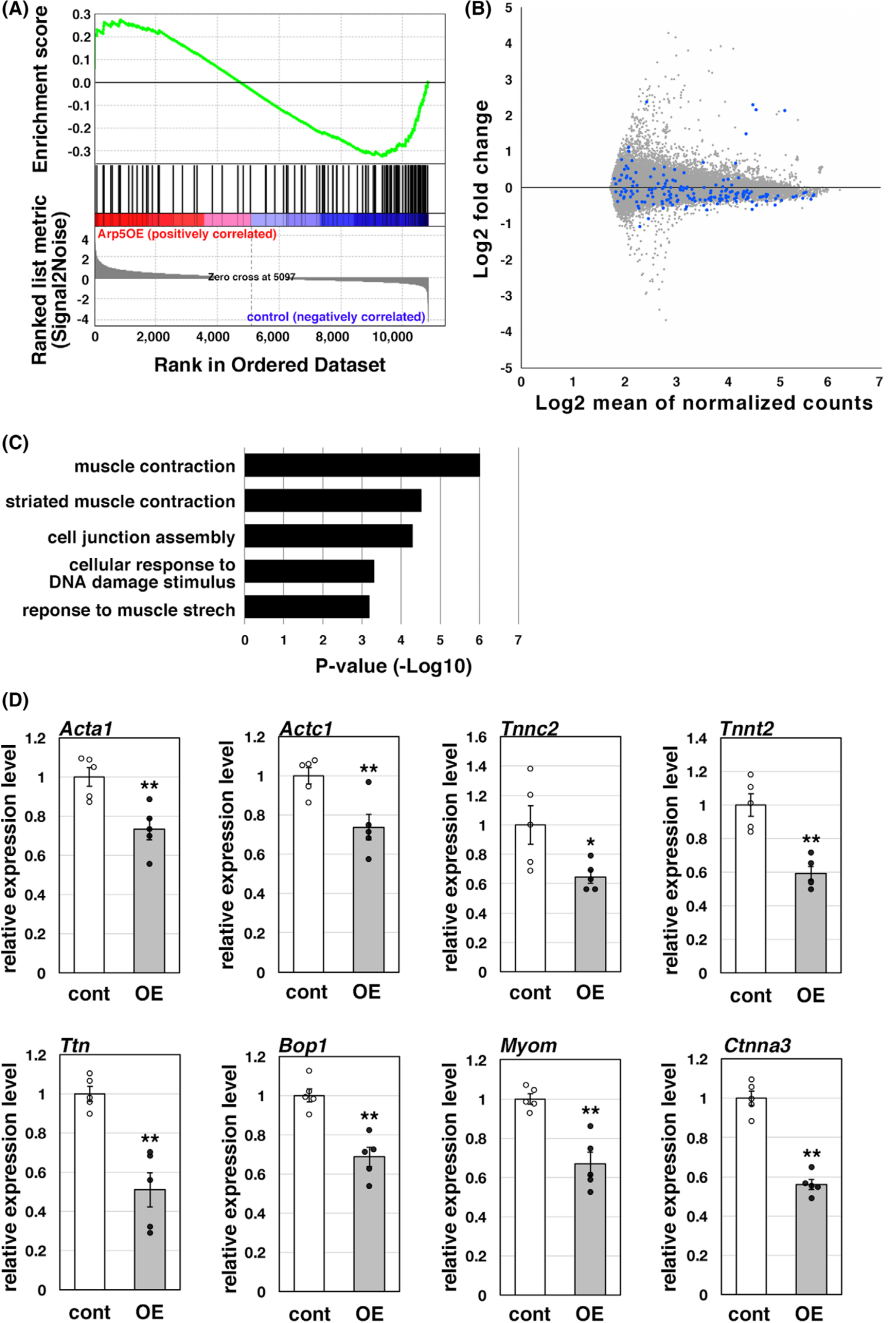
Downregulation of muscle‐related genes in the ARP5‐overexpressing heart. (A) GSEA enrichment plot of the top 1000 genes whose expression was decreased by ARP5‐AAV6 infection in the DNA microarray analysis for gene set DESCARTES_FETAL_MUSCLE_SKELETAL_MUSCLE_CELLS. (B) MA plot of the DNA microarray data between control AAV6‐ and ARP5‐AAV6‐infected hearts. The blue dots indicate the position of the muscle‐related genes. (C) GO analysis of the top 1000 genes whose expression was decreased by ARP5‐AAV6 infection in the DNA microarray analysis. (D) Expression of the muscle‐related genes in control AAV6‐ (cont) and ARP5‐AAV6‐infected (OE) hearts was validated by real‐time RT‐PCR. Statistical data are presented as the mean ± standard error of the mean (SEM). *P < 0.05, **P < 0.01 (Student's *t*‐test).

We also performed GSEA for the top 1000 genes downregulated by ARP5‐AAV6 infection, which revealed the enrichment of muscle‐related genes (Fig. 3A). The expression of 127 out of 176 genes in the gene set ‘DESCARTES_FETAL_MUSCLE_SKELETAL_MUSCLE_CELLS’ was downregulated (Fig. 3B). Gene Ontology (GO) analysis using Metascape [14] also showed that the genes associated with muscle contraction were enriched among the downregulated genes (Fig. 3C). Real‐time RT‐PCR confirmed the decreased expression of muscle‐related genes including *Acta1*, *Actc1*, *Tnnc2*, *Tnnt2*, *Ttn*, *Bop1*, *Myom*, and *Ctnna3* (Fig. 3D). The results suggest that ARP5 plays a role in repressing the expression of muscle‐related genes in cardiomyocytes.

**Fig. 4:**
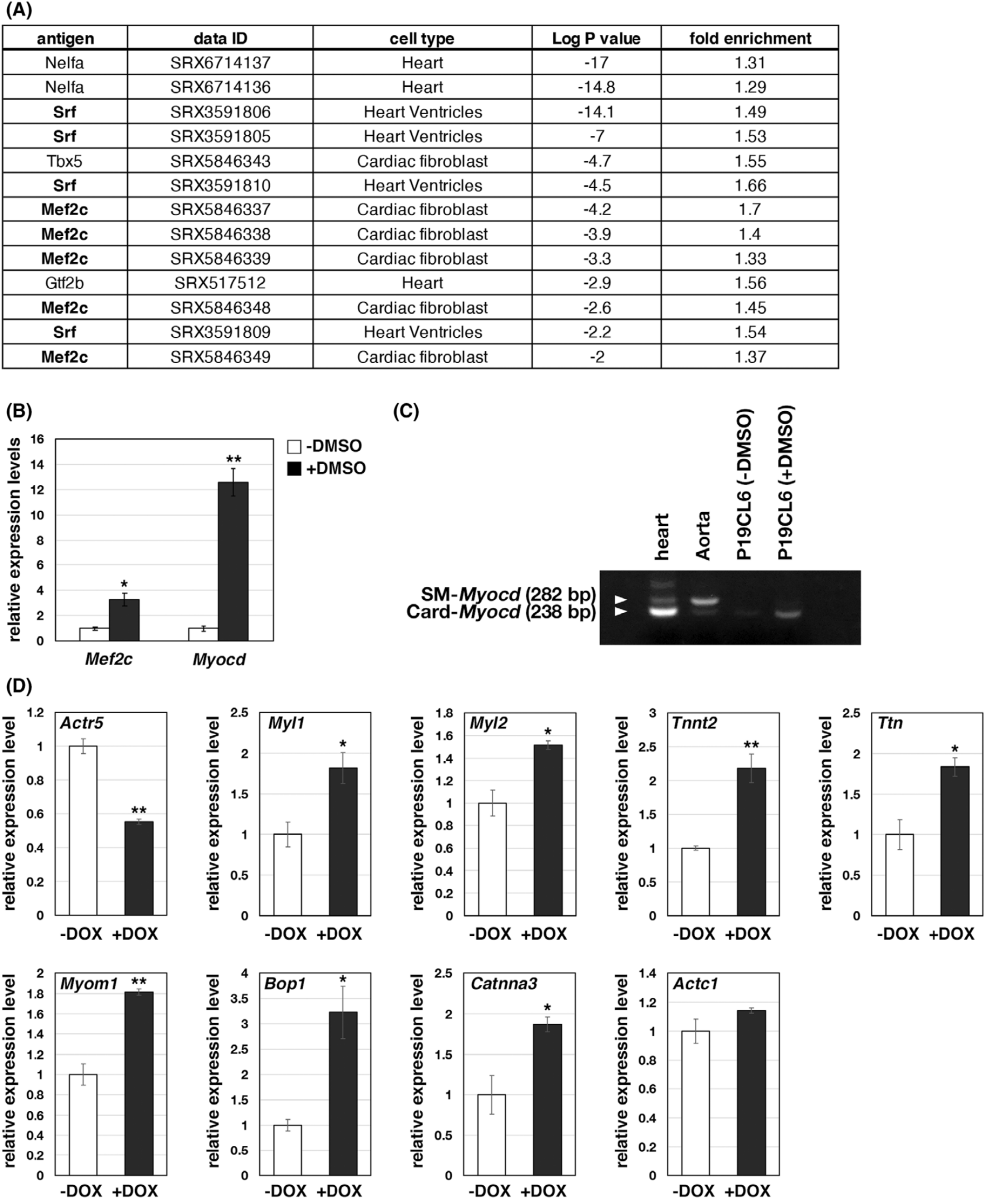
Upregulation of muscle‐related genes by ARP5 knockdown in P19CL6 cells. (A) Enrichment analysis in ChIP Atlas using the top 1000 genes whose expression was decreased by ARP5‐AAV6 infection in the DNA microarray analysis. (B) Real‐time RT‐PCR analysis of *Mef2c* and *Myocd* genes in P19CL6 cells cultured in the presence and absence of DMSO. (C) Identification of the alternative spliced isoform of *Myocd* in murine hearts, aorta, and P19CL6 cells based on the presence or absence of *Myocd* exon 2a by RT‐PCR. White triangles indicate the position of PCR products from smooth muscle type (SM) and cardiac type (Card) isoforms of *Myocd*. (D) Real‐time RT‐PCR analysis of muscle‐related genes in P19CL6 cells transfected with the DOX‐regulated *Actr5* shRNA expression vector. The cells were incubated with DMSO in the presence and absence of DOX for 14 days. All statistical data in (B) and (D) are presented as the mean ± standard error of the mean (SEM). *P < 0.05, **P < 0.01 (Student's *t*‐test).

We previously reported that ARP5 directly binds to the MYOCD–SRF complex and myogenic regulatory factors and inhibits their function in smooth muscle and skeletal muscle cells [5, 10]. To determine which transcription factors regulate the expression of genes downregulated by ARP5‐AAV6 in mouse hearts, the Enrichment analysis tool in ChIP Atlas database [15] was used. This search tool predicts the transcription factors that bind to a particular gene locus using pre‐existing ChIP‐seq data. As expected, SRF was predicted to be recruited to the promoter regions of many of the genes downregulated by ARP5‐AAV6. MEF2C was also predicted to be a central transcription regulator of these genes (Fig. 4A). We examined the role of ARP5 in MYOCD–SRF and MEF2C function using the murine p19 embryonic carcinoma P19CL6 cell line. P19CL6 cells differentiate into cardiomyocytes with cardiac‐specific gene expression when cultured in dimethyl sulfoxide (DMSO) for 1–2 weeks [16]. In the DMSO‐induced cardiomyocyte‐like cells, *Mef2c* and *Myocd* expression was upregulated (Fig. 4B). The *Myocd* gene has cardiac‐specific and smooth muscle‐specific isoforms, and in P19CL6 cells, the cardiac isoform was primarily induced by DMSO treatment (Fig. 4C). For stable doxycycline (DOX)‐regulated knockdown of the *Actr5* gene in P19CL6 cells, cell lines were isolated in which *Actr5*‐targeted shRNA was expressed under the control of the Tet‐On system. When these cells were cultured with DMSO for 14 days in the presence or absence of DOX, DOX maintained *Actr5* expression at approximately half that of the control and significantly increased the expression of cardiac genes including *Myl1*, *Myl2*, *Tnnt2*, *Ttn*, *Myom1*, *Bop1*, and *Catnna3*, which are reportedly regulated by SRF and/or MEF2C (Fig. 4D). *Actc1* expression, which is also induced by SRF, was slightly increased by DOX treatment, but not with statistical significance (Fig. 4D).

### Cooperative action between cardiac MYOCD and MEF2 in cardiac gene expression

**Fig. 5:**
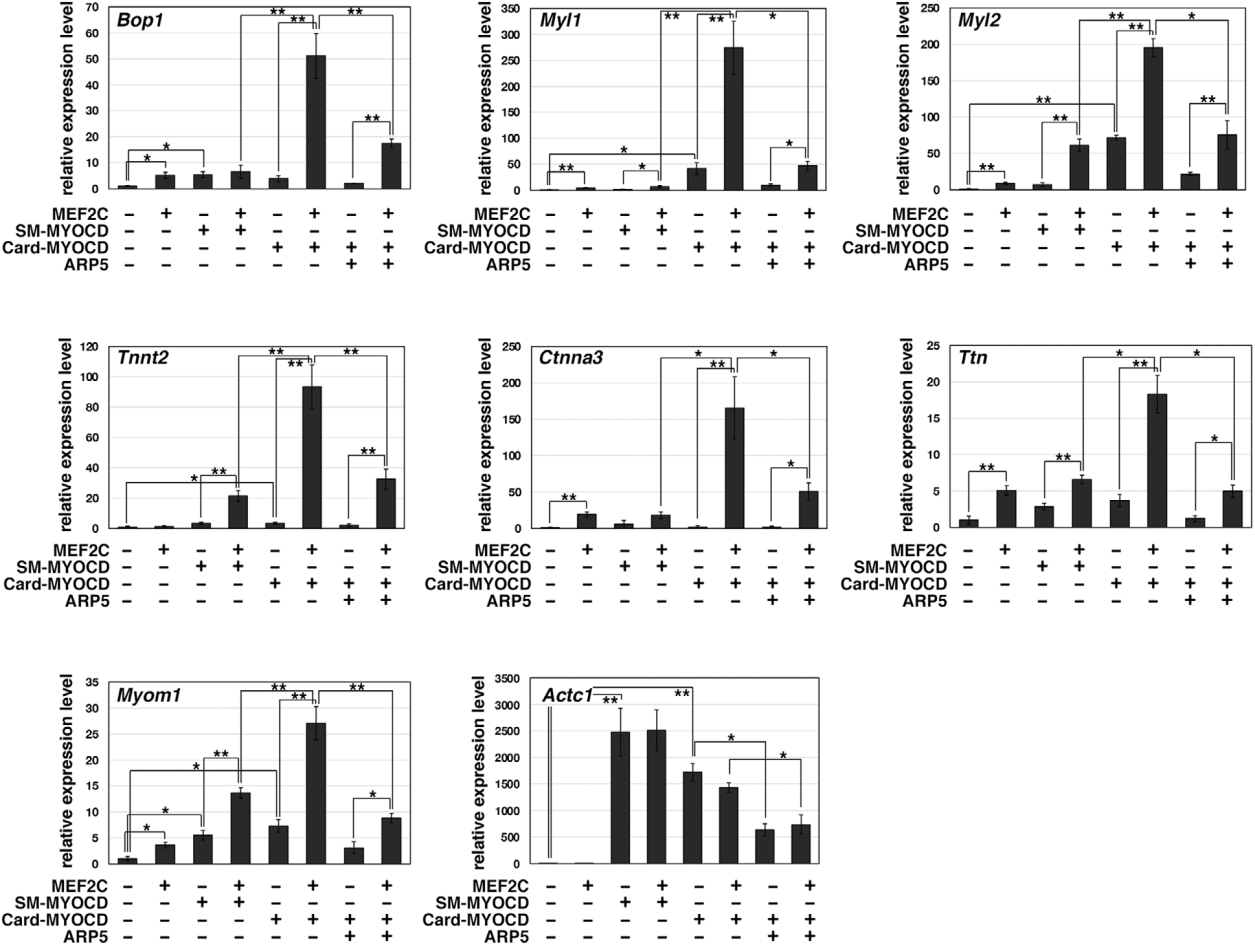
Cooperative action between cardiac MYOCD and MEF2C in muscle‐related gene expression. Real‐time RT‐PCR analysis of muscle‐related genes in P19CL6 cells exogenously expressing MEF2C, smooth muscle type (SM), or cardiac type (Card) isoforms of MYOCD, and ARP5 in the indicated combinations. Statistical data are presented as the mean ± standard error of the mean (SEM). *P < 0.05, **P < 0.01 (Student's *t*‐test).

Creemers et al. [9] previously reported that the cardiac, but not smooth muscle, isoform of MYOCD has a MEF2‐binding motif (MBM) at its N‐terminus and coordinates with MEF2 to activate the transcription of *Myl1*, *Bop1*, and *Srpk3* in an SRF‐independent manner. In P19CL6 cells, MEF2C, cardiac MYOCD, and smooth muscle MYOCD each weakly induced the expression of several cardiac genes. Cardiac MYOCD cooperated with MEF2C to markedly induce the expression of the *Bop1*, *Myl1*, *Myl2*, *Tnnt2*, *Ctnna3*, *Ttn*, and *Myom1* genes, whereas the co‐expression of smooth muscle MYOCD and MEF2C did not show much of a synergistic effect on inducing their expression (Fig. 5). In contrast, the expression of *Actc1* was markedly increased by each isoform of MYOCD alone, without the cooperative action of MEF2C. The excessive expression of ARP5 significantly suppressed the synergistic induction observed with cardiac MYOCD and MEF2C (Fig. 5).

### ARP5 binds near the MEF2‐binding site in the N‐terminus of cardiac MYOCD by competing with MEF2

**Fig. 6:**
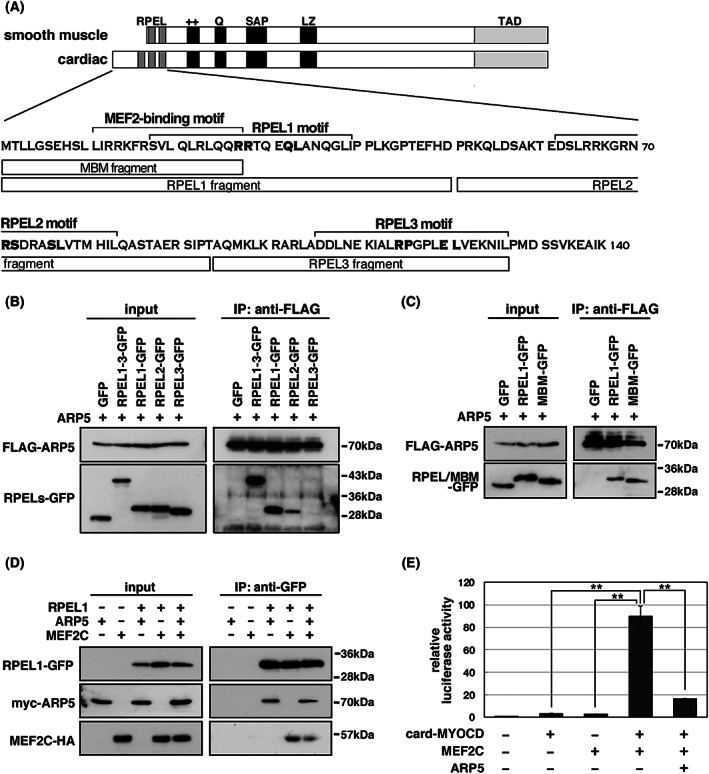
Interaction between ARP5 and distal N‐terminal region of cardiac MYOCD. (A) Schematic diagram of smooth muscle and cardiac MYOCD and the sequence of N‐terminal functional motifs. ++, basic domain; LZ, leucine zipper; Q, glutamine‐rich domain; RPEL, RPEL motifs; SAP, SAP domain; TAD, transactivation domain. Bold letters in the sequence indicate the RPxxxEL residues in the RPEL motifs. The locations of MEF2‐binding motif (MBM) and RPEL fragments used in the co‐immunoprecipitation analysis are also indicated. (B) Co‐immunoprecipitation analysis of FLAG‐tagged ARP5 (FLAG‐ARP5) with GFP‐tagged fragments of the RPEL motifs. (C) Co‐immunoprecipitation analysis of FLAG‐ARP5 with GFP‐tagged fragments of the RPEL1 and MBM. (D) Co‐immunoprecipitation analysis of competitive binding of myc‐ARP5 and MEF2C‐HA to the RPEL1‐GFP. (E) Promoter luciferase assay using the MEF2‐binding element promoter in P19CL6 cells exogenously expressing cardiac MYOCD, MEF2C, and ARP5 in the indicated combinations. Statistical data are presented as the mean ± standard error of the mean (SEM). *P < 0.05, **P < 0.01 (Student's *t*‐test).

Previously, we reported that ARP5 binds to the N‐terminus of MYOCD near the RPEL1 motif [5]. The RPEL1 motif is found only in the cardiac, but not the smooth muscle, isoform of MYOCD, and its sequence overlaps with half of the MBM sequence (Fig. 6A). We confirmed that ARP5 binds strongly to the RPEL1 fragment [amino acid (aa) 1–50 of cardiac MYOCD], faintly to the RPEL2 fragment (aa 51–84), but not to the RPEL3 fragment (aa 85–127), in an immunoprecipitation assay (Fig. 6B). Unexpectedly, the MBM fragment (aa 1–27), which contains the full‐length MEF2‐binding motif, but lacks half of the RPEL1 sequence, maintained sufficient binding to ARP5, similar to that of the RPLE1 fragment (Fig. 6C). This suggests that ARP5, unlike conventional actin, primarily binds near the MBM, rather than RPEL1 motif, at the distal N‐terminus of cardiac MYOCD. In fact, ARP5 interfered with the interaction between RPEL1‐GFP and MEF2C in the immunoprecipitation assay (Fig. 6D). We also performed a promoter luciferase assay with a reporter construct containing four tandem repeats of the MEF2‐binding element (4 × MBE) (Fig. 6E). Because MYOCD itself has no DNA binding capacity, cardiac MYOCD faintly activated the reporter construct. However, the co‐expression of cardiac MYOCD with MEF2C dramatically increased the promoter activity, which was significantly suppressed by ARP5. These results indicate that ARP5 interferes with the interaction between cardiac MYOCD and MEF2, thus preventing their coordinating effect.

### ARP5 binds to the N‐terminus of cardiac MYOCD through its N‐domain

**Fig. 7:**
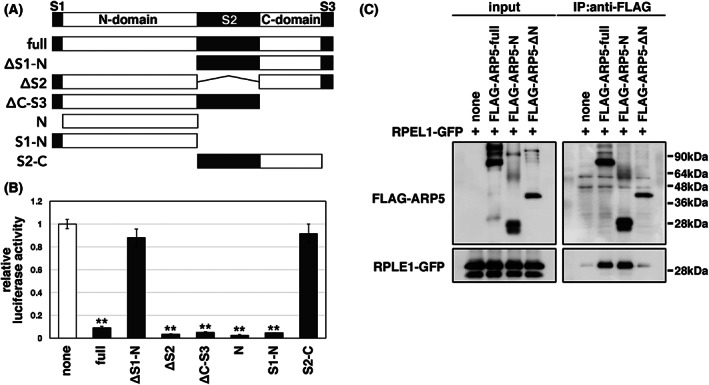
N‐domain requirement for ARP5 to bind to and inhibit cardiac MYOCD. (A) Schematic diagram of the domain‐truncated fragments of ARP5 protein. N‐domain (N) and C‐domain (C) are regions with similarities to other ARPs and conventional actins, while N‐terminal (S1), central (S2), and C‐terminal (S3) regions are unique to ARP5. (B) Promoter luciferase assay using the MEF2‐binding element promoter in P19CL6 cells exogenously expressing cardiac MYOCD and MEF2C together with the indicated truncated ARP5 fragments. Statistical data are presented as the mean ± standard error of the mean (SEM). **P < 0.01 (Student's *t*‐test). (C) Co‐immunoprecipitation analysis of truncated FLAG‐ARP5 fragments with RPEL1‐GFP.

The ARP5 protein consists of N‐domain and C‐domain, which are conserved among conventional actins and other ARPs, and three unique domains in the N‐terminal (S1), central (S2), and C‐terminal (S3) regions (Fig. 7A). We previously reported that the S3 domain is required for ARP5 to bind to SRF and inhibit DNA binding of the MYOCD–SRF complex [5]. The promoter luciferase assay demonstrated that the C‐domain and three unique domains were not required to inhibit cooperative transactivation by cardiac MYOCD and MEF2C, whereas fragments lacking an N‐domain lost the inhibitory activity (Fig. 7B). Furthermore, the N‐domain alone was sufficient to suppress the promoter activity to the same extent as full‐length ARP5 (Fig. 7B). An immunoprecipitation assay also showed that the N‐domain is necessary and sufficient for binding to the N‐terminus of cardiac MYOCD (Fig. 7C). Overall, these data indicate that the N‐domain of ARP5 interacts with the MBM‐containing N‐terminal region of cardiac MYOCD, which, in turn, interferes with the cooperative function of cardiac MYOCD and MEF2.

### Genome‐wide analysis of cardiac gene expression regulated by cardiac MYOCD, MEF2, and ARP5

**Fig. 8:**
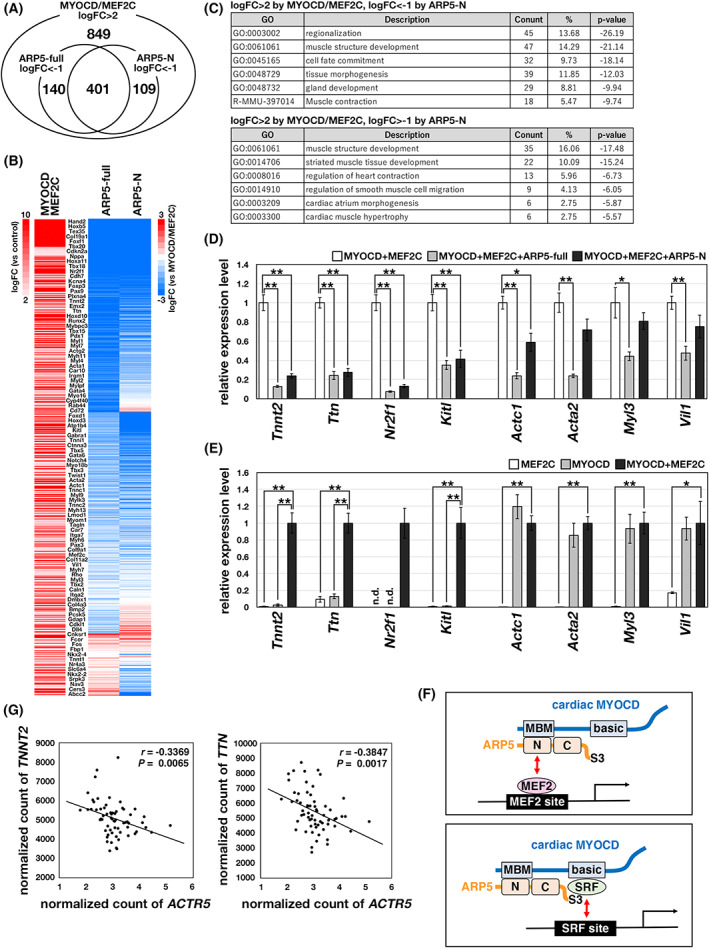
RNA‐seq analysis of cardiac MYOCD‐target genes. (A) Venn diagrams showing the number of genes whose expression was increased more than 4‐fold by the combination of cardiac MYOCD and MEF2C in RNA‐seq analysis. Also shown are the numbers of genes whose increased levels were suppressed by ARP5‐full or ARP5‐N to less than half. logFC represents log2 (fold‐change in gene expression level). (B) Heatmap representing the gene expression profiles from the RNA‐seq analysis. The left panel shows the logFC in the expression levels of the upregulated genes in cardiac MYOCD‐ and MEF2C‐expressing cells relative to mock‐transfected cells (control). The right panels show the logFC in gene expression for the cells expressing ARP5‐full or ARP5‐N in addition to cardiac MYOCD and MEF2C relative to the cardiac MYOCD‐ and MEF2C‐expressing cells. The representative gene names are listed in the center. (C) GO analysis of the genes whose levels of induction by MYOCD and MEF2C were suppressed by ARP5‐N (logFC < −1, upper panel) and those that were not (logFC > −1, lower panel). (D) Real‐time RT‐PCR analysis of cardiac genes in P19CL6 cells exogenously expressing cardiac MYOCD, MEF2C, ARP5‐full, and ARP5‐N in the indicated combinations. (E) Real‐time RT‐PCR analysis of cardiac genes in P19CL6 cells exogenously expressing cardiac MYOCD and/or MEF2C. All statistical data in (D) and (E) are presented as the mean ± standard error of the mean (SEM). *P < 0.05, **P < 0.01 (Student's *t*‐test). (F) Schematics of the inhibitory role of ARP5 in MEF2‐ and SRF‐dependent transactivation of cardiac genes by cardiac MYOCD. (G) Correlation analysis of *ACTR5* expression with *TNNT2* and *TTN* expression in failing human heart samples. RNA‐seq data were obtained from GEO (GSE116250). *r* and *P* indicate Pearson's correlation coefficient and the *P*‐value of Pearson's correlation test, respectively.

To analyze the role of ARP5 in the cardiac MYOCD‐mediated transcriptional regulation on a genome‐wide scale, next‐generation sequencing was done using P19CL6 cells transfected with the full‐length and N‐domain region of ARP5. Based on our previous [5] and present results, full‐length ARP5 (ARP5‐full) inhibits the SRF‐ and MEF2‐mediated transactivation of cardiac MYOCD, whereas the N‐domain of ARP5 (ARP5‐N) inhibits only the MEF2‐mediated transactivation. When cardiac MYOCD and MEF2C were exogenously co‐expressed in P19CL6 cells, the expression of 849 genes was increased more than four‐fold (Fig. 8A,B). Of these, the increased expression of 541 and 510 genes was reduced to less than half by ARP5‐full and ARP5‐N, respectively, and 401 of these suppressed genes overlapped with one another (Fig. 8A,B). A GO analysis revealed that the genes that were strongly induced by cardiac MYOCD and MEF2C and suppressed by ARP5‐N tended to be enriched in GO terms associated with general morphogenetic and differentiation processes, but not limited to muscle development (Fig. 8C, upper panel). In contrast, genes that were not sufficiently suppressed by ARP5‐N were involved in more limited processes related to muscle development and function (Fig. 8C, lower panel). Real‐time RT‐PCR confirmed that the increased expression of *Tnnt2*, *Ttn*, *Nr2f1*, and *Kitl* was suppressed by ARP5‐full and ARP5‐N, whereas that of *Actc1* and *Acta2*, *Myl3*, and *Vil1* was suppressed only by ARP5‐full, but not sufficiently by ARP5‐N (Fig. 8D). The former genes were highly induced through the coordination of cardiac MYOCD and MEF2C, whereas the latter genes were sufficiently induced by cardiac MYOCD alone, independent of MEF2C (Fig. 8E). This supports our hypothesis that ARP5 suppresses cardiac MYOCD activity by interfering with the interaction between cardiac MYOCD and MEF2C through its N‐domain, whereas MEF2‐independent (probably SRF‐dependent) cardiac MYOCD activity is not suppressed by ARP5‐N, which lacks the S3 domain (Fig. 8F). Moreover, from the RNA‐seq data of human dilated and ischemic cardiomyopathy, the expression of *Tnnt2* and *Ttn* was significantly negatively correlated with *Actr5* expression (*r* = −0.3369, *P* = 0.0065 between *Tnnt2* and *Actr5*; *r* = −0.3847, *P* = 0.0017 between *Ttn* and *Actr5*) (Fig. 8G). This suggests that ARP5 is involved in the regulation of cardiac MYOCD‐mediated gene expression under physiological and pathological conditions.

## Discussion

ARP5 is a nuclear‐localized ARP that exhibits a high degree of homology to the conventional actin protein [17, 18]. ARP5 functions as a subunit of the INO80 chromatin remodeling complex in the nucleus without forming filamentous structures [19]. Recently, we reported that ARP5 inhibits smooth and skeletal muscle differentiation in an INO80‐independent manner [5, 10]. ARP5 expression is maintained at relatively low levels in the heart, aorta, and hind limb muscle tissues compared with non‐muscle tissues. ARP5 binds to transcription factors that are required for muscle differentiation, such as MYOCD, SRF, MYOD, and MYOG, and suppresses their transcriptional activities. During smooth muscle cell dedifferentiation and rhabdomyosarcoma development, ARP5 expression is markedly increased, resulting in the decreased expression of various muscle‐specific genes. In the current study, we showed that ARP5 expression was also decreased during murine cardiac development and, conversely, was increased in human cardiomyopathic hearts (Fig. 1A,B). The exogenous overexpression of ARP5 in murine hearts caused cardiac hypertrophy and fibrosis by suppressing the expression of cardiac muscle genes induced by cardiac MYOCD (Figs 1 and 3). The MBM‐containing isoform of MYOCD is expressed also in human cardiovascular tissues, and its expression level is increased in the failing hearts of patients with dilated cardiomyopathy [20, 21]. Furthermore, K259R mutation of MYOCD, which creates a hypomorphic cardiac MYOCD with impaired transactivation capacity, has been reported in patient with congenital heart disease [22]. Thus, MYOCD plays important physiological and pathological roles in the human cardiovascular system. All these suggest that ARP5 plays an inhibitory role in cardiac development and that increased ARP5 in the human heart may contribute to cardiomyopathy and associated cardiac fibrosis through suppression of cardiac MYOCD function.

We previously reported that ARP5 directly binds to the N‐terminal and basic domains of MYOCD, particularly strongly to aa residues 1–50, which contain RPEL1 and MBM sequences [5]. The RPEL motif is a binding site for conventional actin protein [2]. Conserved leucine residues and the RPxxxEL core sequence in the RPEL motif are required for this interaction [4, 23]. Moreover, we previously reported that these leucine residues are not required for the interaction between ARP5 and MYOCD [5]. In the present study, we also demonstrated that the MBM (1–27) fragment lacking half of the RPEL1 motif as well as the RPEL1 (1–50) fragment also bound to ARP5 (Fig. 6C). Thus, ARP5 appears to bind to cardiac MYOCD via a more distal N‐terminal region containing the MBM sequence rather than through the RPEL1 motif. The RPEL motifs are reported to bind to the hydrophobic cleft and ledge formed between subdomains 1 and 3 of G‐actin [23]; however, the hydrophobic residues required for the interaction with the RPEL motif are not conserved in the ARP5 protein (Fig. S1A). An immunoprecipitation and promoter analysis also demonstrated that the N‐domain of ARP5 (ARP5‐N), which includes subdomain 1 and subdomain 2, but not subdomain 3, was sufficient for binding to the N‐terminus of cardiac MYOCD and inhibiting the cooperative action of cardiac MYOCD and MEF2C (Fig. 7). This indicates that ARP5 binds to the N‐terminus of cardiac MYOCD in a manner distinct from conventional actin. AlphaFold is a new deep learning‐based system developed by DeepMind for protein conformation prediction with high accuracy and is comparable to medium‐resolution X‐ray crystallography [24]. It predicted the structure of the RPEL1 domain and β‐actin assembly in good agreement with the previously solved X‐ray crystal structures (Fig. S1B). Notably, this program predicted that the N‐terminus of cardiac MYOCD, particularly the α‐helix formed by aa residues 7–26, interacts with a negatively charged pocket in subdomain 1 of the ARP5 protein (Fig. S1B,C). Overall, these data indicate that the N‐domain of ARP5 interacts near the MBM sequence in the distal N‐terminal region of cardiac MYOCD, thus inhibiting the interaction of cardiac MYOCD with MEF2.

MYOCD has no ability to bind directly to DNA; however, it is recruited indirectly to its target loci by interacting with SRF, which binds to a CArG box DNA cis element^1^. Because SRF binds to both isoforms of MYOCD through their basic and glutamine‐rich domains, CArG box sequences are often found in the promoter and enhancer regions of both cardiac‐ and smooth muscle‐specific genes [25, 26]. By contrast, MEF2 binds to cardiac MYOCD via the MBM motif, which is present only in the cardiac isoform [9]. MEF2, similar to SRF, belongs to the MADS box family of transcription factors and binds to a specific consensus DNA sequence (MEF2‐binding element, MBE) [27, 28, 29], indicating that cardiac MYOCD transactivates multiple cardiac‐restricted promoters in cooperation with MEF2. Based on our previous [5] and present studies, ARP5 plays a dual inhibitory role in the SRF‐ and MEF2‐dependent function of cardiac MYOCD (Fig. 8F). The MEF2 family plays a central role in cardiac development, and they are evolutionary conserved in invertebrate and vertebrate organisms [30]. Furthermore, the combination of transcription factors MEF2C, GATA4, and TBX5 induces direct cardiac reprogramming of mouse fibroblast cells [31], and MYOCD facilitates their reprogramming efficiency [32]. Thus, MEF2 and MYOCD are important transcription factors for cardiac gene expression; however, little is known regarding the significance of their cooperative action through their physical interaction. Creemers et al. found that the expression of *Myl1*, *Bop1*, and *Srpk3* is synergically regulated by cardiac MYOCD and MEF2C [9]. In the present study, we identified several cardiac genes that are markedly induced by cardiac MYOCD in concert with MEF2C, but not by smooth muscle MYOCD (Fig. 5). Moreover, RNA‐seq analysis demonstrated that the expression of 849 genes was upregulated by the combination of cardiac MYOCD and MEF2C, of which 510 were suppressed by ARP5‐N (Fig. 8A,B). This suggests that cardiac MYOCD promotes the transcription of many cardiac genes by binding to MEF2 and ARP5 negatively modulates this cooperative action by interfering with their physical interaction.

Huang et al. [33] found that conditional ablation of the *Myocd* gene in the adult mouse heart results in marked cardiac enlargement and cardiomyocyte disarray with myocyte loss and fibrosis. In MYOCD‐defective hearts, the expression of MYOCD‐targeted myofibrillar structural genes, such as alpha‐cardiac actin, alpha‐myosin heavy chain, myosin light chain 2v, tropomyosin, alpha‐actinin, and desmin, was reduced by 80–90%, and cardiomyocyte apoptosis was frequently observed. These phenotypes were similar to that observed in ARP5‐AAV6‐infected hearts including heart enlargement, fibrosis, and, to a lesser extent, downregulation of genes associated with muscle structure and function (Figs 1 and 3). This may be due to the repression of MYOCD activity by the modest overexpression of ARP5, which was much milder than that occurring by *Myocd* ablation. In P19CL6 cells, the cooperative activity of MEF2 and cardiac MYOCD was reduced to 20% through ARP5 overexpression in a cell‐autonomous fashion (Figs 7B and 8D), and the expression of endogenous cardiac genes was significantly increased by ARP5 knockdown (Fig. 4D). This suggests that the abnormalities in ARP5‐overexpressing hearts result from the suppression of cardiac MYOCD function.

In summary, ARP5 binds near the N‐terminal MEF2‐binding region of cardiac MYOCD and inhibits the cooperative action of cardiac MYOCD with MEF2 in cardiomyocytes. Excessive expression of ARP5 leads to dysregulation of cardiac MYOCD‐mediated gene expression, resulting in cardiac hypertrophy and fibrosis. This also raises the possibility that elevated ARP5 expression may contribute to cardiomyopathy. The major limitation of this study is the lack of data analyzing the role of ARP5 in the physiological function of the heart. In addition, although AAV6 is most efficiently introduced into the heart, the method of ARP5 overexpression by AAV6 has not eliminated the indirect effects of AAV6 infection in the organs other than the heart. Further experiments, such as conditional tissue‐specific knockout and overexpression of ARP5 in the heart, are needed to fully elucidate the significance of ARP5 in cardiac development and function. Nonetheless, our data provide new insights into the cardiac developmental and pathological roles of ARP5 in the regulation of cardiac gene expression by MYOCD and also provide a better understanding of the cardiac‐specific function of MYOCD through MEF2 binding. Furthermore, since both MYOCD and MEF2 are central transcriptional regulators in direct cardiac reprogramming, the suppression of ARP5 function is expected to increase the conversion efficiency of cells with high ARP5 expression into cardiomyocytes.

## Materials and methods

### Regents and antibodies

DMSO, G418, and doxycycline hyclate (DOX) were purchased from Nacalai Tesque (Kyoto, Japan), Adipogen Life Sciences (Liestal, Switzerland), and Cayman Chemical Company (Ann Arbor, MI, USA), respectively. The following antibodies were used: anti‐FLAG (Sigma‐Aldrich, St. Louis, MO, USA), anti‐HA (Roche Applied Science, Penzberg, Germany), anti‐Myc (Santa Cruz Biotechnology, Inc, Dallas, TX, USA), anti‐Arp5 (Proteintech, Rosemont, IL, USA), anti‐GAPDH (Thermo Fisher Scientific, Waltham, MA, USA), anti‐ACTA2 (Sigma‐Aldrich), anti‐Collagen I (Novus Biologicals, Centennial, CO, USA), and anti‐GFP (Proteintech) antibodies.

### Plasmids and AAV expression vector

The coding regions of human *ACTR5*, the cardiac and smooth muscle isoforms of mouse *Myocd*, and *Mef2c* were amplified by PCR and inserted into the pCAGGS and pCS2+ expression vectors. A tetracycline‐inducible shRNA vector, pSingle‐tTs‐shRNA (Clontech), was used to isolate cell lines exhibiting *Actr5* knockdown under DOX control. The target sequence for the *Actr5* knockdown was gacagatggaccagtttca, and the hairpin loop sequence was ttcaagaga. The AAV6 expression vector encoding *Actr5* was constructed using the AAVpro Helper Free System (AAV6) (TAKARA BIO, Shiga, Japan). Purification and titration of AAV6 particles were performed using the AAVpro Purification Kit Maxi (All Serotypes) (TAKARA BIO) and AAVpro titration (TAKARA BIO) kits.

### Cell cultures and transfection

P19CL6 cells (supplied by RIKEN BRC Cell Bank) were cultured in Minimum Essential Medium Eagle Alpha Modification containing l‐glutamine and phenol red (FUJIFILM Wako Pure Chemical Corporation, Osaka, Japan) and supplemented with 10% Tet‐system approved FBS (Thermo Fisher Scientific). P19CL6 cell lines expressing *Actr5* shRNA were cultured with 400 μg·mL^−1^ of G418 to maintain the drag‐resistant phenotype. To differentiate P19CL6 cells into cardiomyocytes, the cells were plated on a gelatin‐coated culture dish and incubated in Dulbecco's Modified Eagle's Medium containing l‐glutamine and phenol red (FUJIFILM Wako Pure Chemical Corporation) supplemented with 8% FBS, 1% DMSO, and 1 μg·mL^−1^ DOX for 14 days, with a change of medium every 2 days. All cell transfection experiments were performed using Lipofectamine 3000 (Thermo Fisher Scientific).

### Animal studies

All animal experiments were conducted in accordance with the animal experimental guidelines established by the Osaka University School of Medicine, Japan. The mice were housed under specific pathogen‐free conditions at ~ 22 °C with a 12‐h cycle of light and dark. Food and water were supplied *ad libitum*. The protocol was approved by the animal experiments committee of Osaka University with the approval number 24‐004‐003.

For ARP5 overexpression in murine hearts, 1 × 10^9^ gv·μL^−1^ of control‐AAV6 (empty vector) or ARP5‐AAV6 vector was intraperitoneally injected into 3‐week‐old female C57BL/6J mice. Three weeks after injection, the mice were sacrificed by intraperitoneal administration of 200 mg·kg^−1^ sodium pentobarbital to ameliorate suffering. The hearts were then extracted, fixed in 10% formalin, embedded in paraffin, and cut into 5‐μm‐thick sections. The sections were stained with hematoxylin and eosin and observed by microscopy. Cardiac fibrosis was assessed by Masson's trichrome staining.

### Real‐time RT‐PCR

Total RNA was isolated using RNAIso Plus (TAKARA BIO) and NucleoSpin RNA plus (MACHEREY‐NAGEL, Düren, Germany) and reverse‐transcribed using PrimeScript RT Reagent Kit with gDNA Eraser (TAKARA BIO). Real‐time RT‐PCR was done using a Mic real‐time PCR cycler (Bio Molecular System) with THUNDERBIRD SYBR qPCT Mix (TOYOBO, Osaka, Japan). The nucleotide sequences of the primer sets are listed in Table S1.

### Western blotting

Western blotting was performed as described previously with minor modifications [34]. Briefly, the proteins in 2% sodium dodecyl sulfate sample buffer were electrophoretically separated using 7.5% or 10% polyacrylamide gel and then transferred to polyvinylidene difluoride membrane. The membrane was incubated with the blocking reagent Blocking One (Nacalai Tesque) and then incubated with a primary antibody. After washing, the membrane was incubated with horseradish peroxidase‐conjugated secondary antibody. Chemiluminescence detection was performed using Chemi‐Lumi One Ultra (Nacalai Tesque). The band intensities of proteins of interest were quantified using the imagej software [35], which were normalized by GAPDH intensity.

### Promoter luciferase assay

Four tandem repeats of the MEF2‐binding element (tcggactgttaCTAAAAATAGcacacctg) were inserted into the pGL3‐basic vector (Promega) with the *Xenopus* γ‐actin‐TATA sequence [34]. This reporter vector was transfected into P19CL6 cells along with the indicated gene expression vectors and the pSV‐βGal vector (Promega). Two days after transfection, luciferase activity was measured using the Luciferase Assay System (Promega), which was normalized by β‐galactosidase activity.

### Co‐immunoprecipitation

Recombinant proteins were synthesized *in vitro* using the TNT SP6 High‐Yield Wheat Germ Protein Expression System (Promega) with the gene‐inserted pCS2+ vectors. The synthesized proteins were diluted in a binding buffer [0.1% NP‐40, 10% glycerol, 50 mm imidazole, protease inhibitor cocktail (Nacalai Tesque) in 0.5× PBS] and incubated with FLAG‐Affinity Gels (Sigma‐Aldrich) for 6 h. The resulting beads were washed three times with a wash buffer (1% Triton X‐100, 0.1% sodium deoxycholate, 150 mm KCl, and 20 mm Tris–HCl, pH 6.8), and the bound proteins were eluted with SDS‐containing sample buffer. The eluted proteins were electrophoretically separated by SDS/PAGE and then visualized by western blot analysis with the indicated antibodies.

### DNA microarray

Total RNA was extracted and purified from three control‐AAV6‐infected and three ARP5‐AAV6‐infected hearts. These biological replicate RNA samples were mixed in equal amounts and used for microarray analysis. The mRNAs were reverse‐transcribed and Cy3‐labeled using the Low Input Quick Amp Labeling kit (Agilent Technologies). The labeled complementary RNAs were hybridized onto a SurePrint G3 Mouse GE 8 × 60 K v2 Microarray (Agilent Technologies). Fluorescence signals were detected using the SureScan Microarray Scanner (Agilent Technologies), and the intensities were quantified using feature extraction software (Agilent Technologies, Santa Clara, CA, USA). GSEA was performed using gsea software (v4.2.2) [12, 13].

### RNA sequencing

Total RNAs were extracted from P19CL6 cells exogenously expressing cardiac MYOCD, MEF2C, ARP5‐full, or ARP5‐N in the indicated combinations. RNAs from three independent experiments were mixed in equal amounts and purified using the NEBNext Poly(A) mRNA Magnetic Isolation Module (for PolyA selection) (New England Biolabs). RNA libraries were constructed using the NEBNext Ultra II Directional RNA Library Prep Kit (New England Biolabs, Ipswich, MA, USA) and sequenced using 150 bp paired‐end reads on a NovaSeq 6000 (Illumina, San Diego, CA, USA). A quality check of the raw sequence data was performed using fastqc (v0.11.8‐0). The resulting data were mapped onto the mouse reference GRCm39 using an RNA‐seq read mapper star (v2.7.10a). The expression levels of the genes and isoforms were estimated using a software tool for the quantification of RNA‐seq data RSEM. GO analysis was performed using the Metascape [14] gene annotation and analysis resource. For genes with an FPKM‐normalized count of 0 in the control cells, if their counts in the MYOCD‐MEF2C‐expressing cells were 5 or higher, they were considered to be sufficiently induced by MYOCD‐MEF2C.

### Statistics and reproducibility

All statistical data in this study were generated from at least three repetitions of independent biological experiments. Data were evaluated by a Student's *t*‐test with *P < 0.05 and **P < 0.01 considered as statistically significant. For the DNA microarray and RNA sequence analyses, three biological replicate RNA samples were mixed and used for a subsequent analysis to determine the average of three samples. For western blot and histological analyses, representative images were selected from at least three independent experiments.

## Conflict of interest

The authors declare that there is no conflict of interest.

## Author contributions

TM and KH involved in conceptualization and design of the study; KH involved in the development of methodology; TM involved in the acquisition of the data; TM and KH involved in the analysis and interpretation of the data; TM involved in writing of the manuscript; TM and KH involved in study supervision; all authors read and approved the final manuscript.

## Supporting information


**Fig. S1.** Differential MYOCD interaction between β‐actin and ARP5. (A) Sequence alignment of the hydrophobic cleft and ledge region of β‐actin (ACTB) with the corresponding region of ARP5. Asterisks indicate the residues conserved between ACTB and ARP5. Arrows and red letters indicate the predominantly hydrophobic and RPEL‐interacting residues in ACTB, respectively. (B) *In silico* predicted structures of ACTB (green, left panel) and ARP5 (green, right panel) complexed with the N‐terminal fragment of cardiac MYOCD (amino acids 1 to 60, yellow). These structures were generated using the AlphaFold2‐multimer program in the ColabFold notebook. (C) Electrostatic surface potential map of ARP5 in a complex with the N‐terminal fragment of cardiac MYOCD. Adaptive Poisson‐Boltzmann Solver electrostatic potential was calculated using CueMol software (v2.2.3.443). Positive and negative potentials are shown in blue and red, respectively.

**Table S1.** List of PCR primer sequences used in this study.

## Data Availability

DNA microarray data are available in the GEO database (https://www.ncbi.nlm.nih.gov/geo/query/acc.cgi?acc=GSE197087) under the accession number GSE197087. RNA sequence data are available in the DDBJ database (https://ddbj.nig.ac.jp/resource/bioproject/PRJDB14073) under the run numbers DRR395966–DRR395969. The publicly available RNA‐seq data in Fig. 1A (accession number GSE116250 and GSE46224), 1B (GSE116250 and GSE46224), and 8G (GSE116250) were obtained from the GEO database.
