# Supplementary material for: Actin‐related protein 5 suppresses the cooperative activation of cardiac gene transcription by myocardin and MEF2
Source: FEBS Open Bio. 2023 Jan 12;13(2):363–79. doi: 10.1002/2211-5463.13549 (PMC9900090; doi:10.1002/2211-5463.13549)

A

```
                          ↓  ↓                       ↓↓ ↓ ↓
ACTB   FETFNTPAMYVAIQAVLSLYASG----RTTGIVMDSGDGVTHTVPIYEGYAL 171
ARP5   FECYGIPKVAYGIDSLFSFYHNKPKNSMCSGLIISSGYQCTHVLPILEGRLD 205
       **    *     *    * *          *    **   **  ** **

                  ↓↓  ↓ ↓   ↓                   ↓
ACTB   RKYSVWIGG-SILASLSTFQQMWISKQEYDESGPSIVHRKCF 375
ARP5   PVLDAWYGARDWALNHLDDNEVWITRKEYEEKGGEYLKEHCA 560
            * *               **   **   *       *
```

B

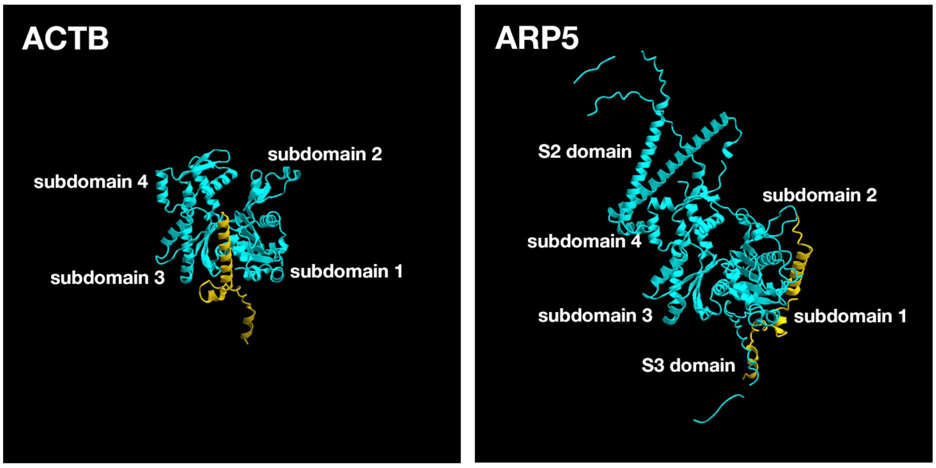

C

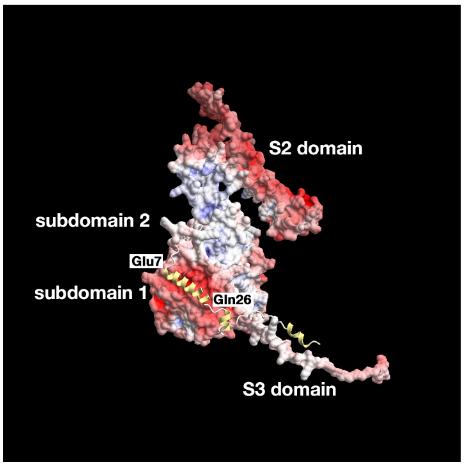

Supplement: Supplementary file 1 — Fig. S1. Differential MYOCD interaction between β‐actin and ARP5. (A) Sequence alignment of the hydrophobic cleft and ledge region of β‐actin (ACTB) with the corresponding region of ARP5. Asterisks indicate the residues conserved between ACTB and ARP5. Arrows and red letters indicate the predominantly hydrophobic and RPEL‐interacting residues in ACTB, respectively. (B) In silico predicted structures of ACTB (green, left panel) and ARP5 (green, right panel) complexed with the N‐terminal fragment of cardiac MYOCD (amino acids 1 to 60, yellow). These structures were generated using the AlphaFold2‐multimer program in the ColabFold notebook. (C) Electrostatic surface potential map of ARP5 in a complex with the N‐terminal fragment of cardiac MYOCD. Adaptive Poisson‐Boltzmann Solver electrostatic potential was calculated using CueMol software (v2.2.3.443). Positive and negative potentials are shown in blue and red, respectively. [file FEB4-13-363-s001.pdf]
